# Crystal structure of 4-(3,5-dimethyl-1,7-diphenyl-1,7-di­hydro­dipyrazolo­[3,4-*b*:4′,3′-*e*]pyridin-4-yl)phenol dimethyl sulfoxide disolvate

**DOI:** 10.1107/S2056989025006152

**Published:** 2025-07-23

**Authors:** N’gandi N’gaza, Erwann Jeanneau, Catherine Marestin, Regis Mercier

**Affiliations:** a Universite Claude Bernard Lyon1 Université Jean Monnet CNRS UMR 5223 Ingénierie des Matériaux Polymères F69 621 Cédex France; b Centre de Diffractométrie Henrin Longchambon Universite Claude Bernard Lyon1 69100 Villeurbanne France; University of Massachusetts Dartmouth, USA

**Keywords:** crystal structure, di­hydro­dipyrazolo­pyridine

## Abstract

A new bis­pyrazolyl­pyridine­(BPP)-containing derivative, namely, 4-(3,5-di­meth­yl-1,7-diphenyl-1,7-di­hydro­dipyrazolo­[3,4-*b*:4′,3′-*e*]pyridin-4-yl)phenol (**1**), C_27_H_21_N_5_O·2C_2_H_6_OS, has been synthesized by a Hantzsch multicomponent reaction using acetic acid (AcOH) as solvent and copper(II) acetate monohydrate [Cu(OAc)_2_·H_2_O] as mild oxidant. The structure of the compound was fully characterized by single-crystal X-ray diffraction. The crystallographic study shows that the mol­ecule crystallizes in the triclinic system with space group *P*1, with one mol­ecule per asymmetric unit. Whereas the phenolic substituent in the *para* position of the pyridine group is highly twisted from the planar bis­pyrazolo­pyridine core [56.19 (4)°], the two phenyl rings present on the nitro­gen atoms are only slightly distorted [11.77 (5) and 29.17 (5)°]. The crystal structure is consolidated by inter­molecular C—H⋯O hydrogen bonds and π–π stacking inter­actions.

## Abstract 2

The mol­ecular and crystal structure of the novel compound 4-(3,5-Dimethyl-1,7-diphenyl-1,7-di­hydro­dipyrazolo­[3,4 − b:4′,3′-e]pyridin-4-yl)phenol are reported.

## 1. Chemical context

Bis-pyrazolo­[3,4-*b*:4′,3′-e]pyridine (BPP) is a tricyclic scaffold known for its strong fluorescence, making its derivatives valuable as light-emitting materials in electroluminescent devices (Safaei-Ghomi *et al.*, 2016 ▸; Ko & Tao, 2001 ▸). BPP-containing derivatives are usually synthesized by pseudo three-component reaction between 5-amino­pyrazole and aromatic aldehydes at 493–523 K. Such experimental conditions result in low to moderate yields (Gondek *et al.*, 2012 ▸; Puchala *et al.*, 1997 ▸). Alternative methods include microwave-assisted, solvent-free conditions (Quiroga *et al.*, 2005 ▸), ionic liquid-mediated synthesis (Shi & Yang, 2008 ▸), and FeCl_3_ catalysis in DMSO at 403 K (Qiu *et al.*, 2018 ▸).
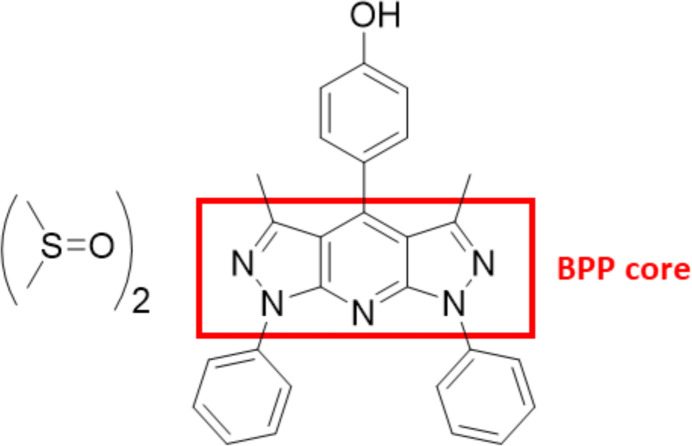


As part of our ongoing work related to the synthesis of nitro­gen-containing heterocycles, we synthesized the title compound **1** via oxidative multicomponent condensation using acetic acid (AcOH) as a solvent and copper(II) acetate monohydrate [Cu(OAc)_2_·H_2_O] as a mild oxidant for di­hydro­pyridine derivatives (Bell & Rothenberger, 1987 ▸; Qiu *et al.*, 2018 ▸). In such conditions, analytically pure phenol-containing bis­pyrazolo­pyridine was isolated with 70% yield.

## 2. Structural commentary

**Figure 1:**
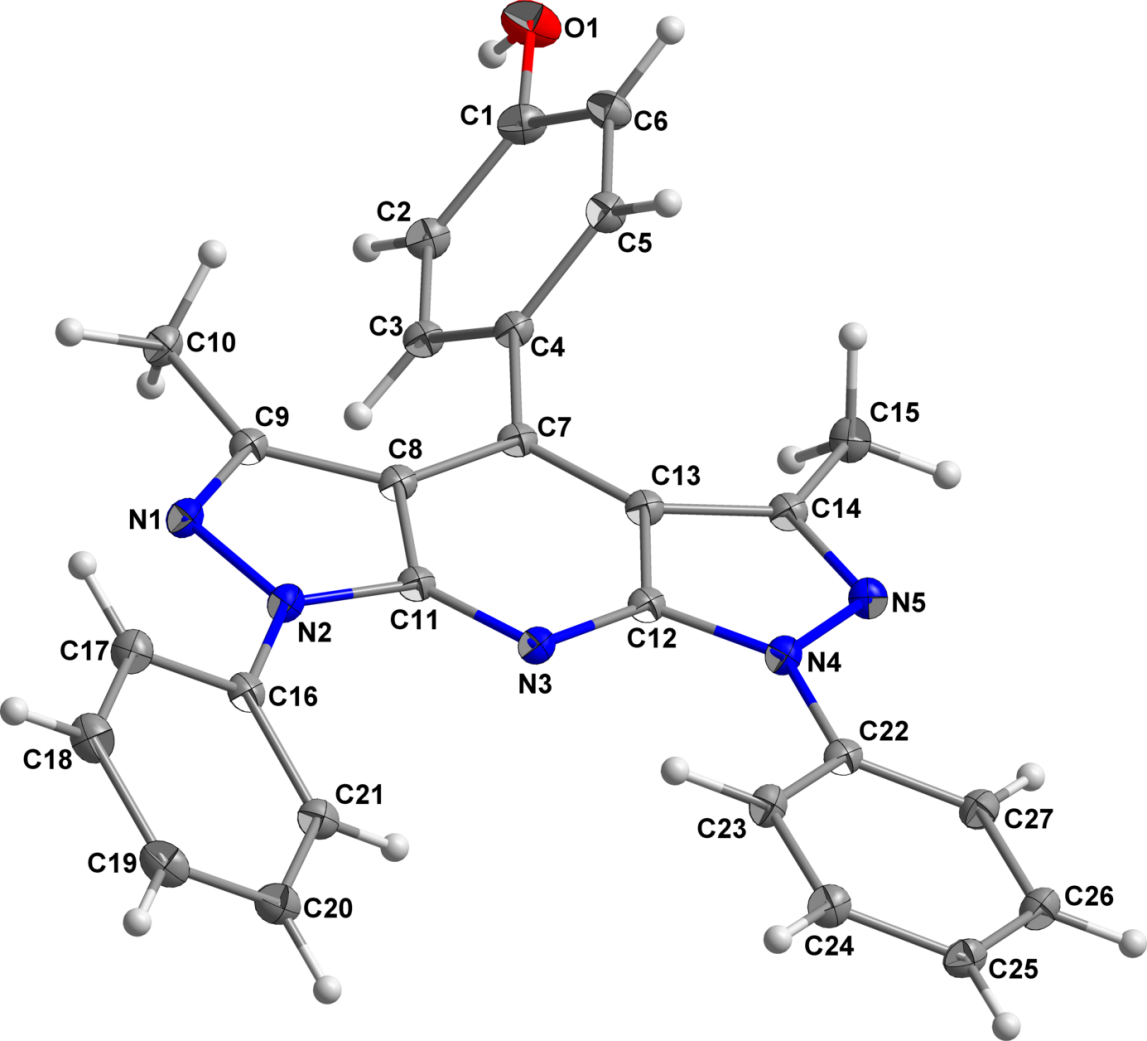
Mol­ecular view of the title compound with displacement ellipsoids drawn at the 30% probability level (DMSO solvent mol­ecules are omitted for clarity).

The asymmetric unit of the title compound is composed of a 4-(3,5-dimethyl-1,7-diphenyl-1,7-di­hydro­dipyrazolo­[3,4-*b*:4′,3′-*e*]pyridin-4-yl)phenol mol­ecule and two crystallographically distinct DMSO mol­ecules. The twelve-membered fused-ring system is essentially planar and symmetrical about the N3⋯C7 line (Fig. 1 ▸). The r.m.s. deviation of the twelve atoms from the mean plane is 0.035 Å. The dihedral angles between the phenyl rings and the mean plane of the twelve-membered fused-ring system are 11.77 (5) and 29.17 (5)° for the ring defined by atoms C22–C27 and the ring defined by atoms C16–C21, respectively. The dihedral angle between the twelve-membered ring and the phenolic substituent is much greater with a value of 56.19 (4)°, due to the steric hindrance of the two methyl groups. This BPP derivative co-crystallizes with two DMSO mol­ecules, one of which exhibits a slight positional disorder of the sulfur atoms over two crystallographic positions with occupancies of 0.923 (2) and 0.077 (2).

## 3. Supra­molecular features

**Figure 2:**
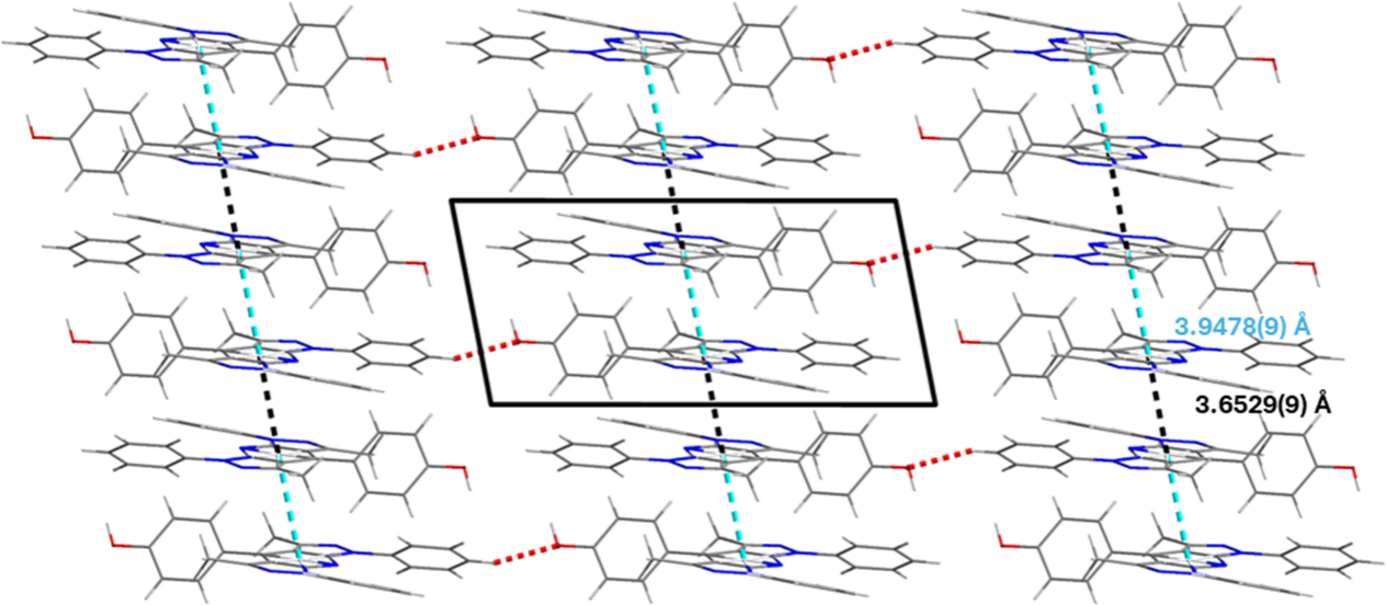
View of the packing of the title compound with inter­molecular inter­actions represented as dotted lines (black and blue: –π stacking, red: C—H⋯O hydrogen bonding.

The BPP-derivative mol­ecules lie nearly perpendicular to the *a* axis of the unit cell and are related to one another through an inversion center. This leads to chains where the mol­ecules are arranged in a head-to-tail manner with two distinct inter­planar distances (Fig. 2 ▸). The distance of 3.9478 (9) Å corresponds to π–π inter­actions between two adjacent pyridine rings whereas the shorter distance of 3.6529 (9) Å is probably due to a combination of the pyridine rings π–π inter­action reinforced by an inter­action between the hydrogen atoms from the phenyl groups and the pyrazole rings. These infinite chains are linked together along the *c-*axis direction by C—H⋯O hydrogen-bonding inter­actions (Table 1 ▸).

## 4. Database survey

A structural fragment search for the twelve-membered fused-ring subsituted with two phenyl rings in the Cambridge Structural Database (CSD version 5.46, last update November 2024; Groom *et al.*, 2016 ▸) resulted in five hits. One hit corresponds to the structure of the unsubstituted fragment (ADAJAR; Portilla *et al.*, 2006 ▸) and the others correspond to the similar fused-ring system with different substituents: a phenyl ring (FEPDEJ; Krygowski *et al.*, 1998 ▸; FEPDEJ01; Low *et al.*, 2003*b* ▸), phenylNMe2 (FEPDUZ; Krygowski *et al.*, 1998 ▸), a pyridin (IKIFEN; Low *et al.*, 2003*a* ▸ and a COPh (KEQRAC; Gao *et al.*, 2018 ▸). While the two phenyl rings in the unsubstituted compound (ADAJAR) both have a dihedral angle of 27.9 (3)° with respect to the BPP core, the title compound displays one small angle of 11.77 (5)° and a larger one of 29.17 (5)°. This feature is also found for most of the substituted referenced compounds [FEPDEJ01: 5.00 (3) and 26.49 (3)°; FEDPUZ: 8.23 (6° and 34.65 (6)°; IKIFEN: 7.11 (3) and 22.8 (3)°] with the exception of KEQRAC where the phenyl rings both make a small dihedral angle with respect to the BPP core [7.38 (8) and 8.12 (6)°]. Similarly, the angles between the BPP core and the substituent located opposite the phenyl rings lie in a range of about 10° [FEPDEJ01: 62.92 (3); FEDPUZ: 67.94 (5)°; IKIFEN: 70.46 (3)°]. The title compound lies in the bottom range with a dihedral angle of 56.19 (4)° while KEQRAC displays an almost right angle [*ie.*. 89.19 (5)°] between its substituent and the BPP core.

## 5. Synthesis and crystallization

A 50 mL single-neck round-bottom flask equipped with a magnetic stirring bar and a condenser was charged with 3-methyl-1-phenyl-1*H*-pyrazol-5-amine (11.5 mmol, 2 eq), benzaldehyde (5.7 mmol, 1 eq), and Cu(OAc)_2_·H_2_O (2.8 mmol, 0.5 eq) in 20 mL of AcOH. Tri­ethyl­amine (1 mL) was added, and the reaction mixture was heated at 403 K for 48 h under continuous stirring. Reaction progress was monitored by thin-layer chromatography (TLC) (DCM/Petroleum ether, 70:30 *v*/*v*). Upon completion, the reaction mixture was precipitated in 200 mL of demineralized water, and the crude solid was collected by filtration. The solid was dissolved in 1,3-dioxolane. The resulting mixture was charcoal-washed, filtered through a Celite pad, and the solvent was evaporated. The product was purified via column chromatography (DCM/petroleum ether, 3:1 *v*/*v*), yielding a pure white solid. 70% yield [m.p. 558 K; lit. 430–431 K (Hennig *et al.*, 1990 ▸), 500–501, (Shi *et al.*, 2008 ▸)]. Recrystallization from dimethyl sulfoxide (DMSO) gave single crystals suitable for X-ray diffraction analysis.

## 6. Refinement

Crystal data, data collection and structure refinement details are summarized in Table 2 ▸. One of the two DMSO solvent mol­ecules was slightly disordered over two positions. SADI restraints were used for this mol­ecule in the course of the refinement. H atoms were positioned geometrically and refined as riding [C—H = 0.5–0/98 Å, *U*_iso_(H) = 1.2–1.5*U*_eq_(C)].

## Supplementary Material

Crystal structure: contains datablock(s) I. DOI: 10.1107/S2056989025006152/yy2018sup1.cif

Structure factors: contains datablock(s) I. DOI: 10.1107/S2056989025006152/yy2018Isup3.hkl

Supporting information file. DOI: 10.1107/S2056989025006152/yy2018Isup3.cml

CCDC reference: 2472025

Additional supporting information:  crystallographic information; 3D view; checkCIF report

## Figures and Tables

**Table 1:** Hydrogen-bond geometry (Å, °)

*D*—H⋯*A*	*D*—H	H⋯*A*	*D*⋯*A*	*D*—H⋯*A*
C19—H19⋯O1^i^	0.95	2.37	3.283 (2)	160
C29—H29*B*⋯O2^ii^	0.98	2.48	3.312 (3)	142

Symmetry codes: (i) 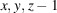; (ii) 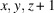.

**Table 2:** Experimental details

Crystal data	
Chemical formula	C_27_H_21_N_5_O·2C_2_H_6_OS
*M*_r_	587.74
Crystal system, space group	Triclinic, *P* 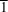
Temperature (K)	100
*a*, *b*, *c* (Å)	7.5736 (1), 12.0242 (1), 16.1594 (2)
α, β, γ (°)	91.824 (1), 100.953 (1), 92.657 (1)
*V* (Å^3^)	1441.98 (3)
*Z*	2
Radiation type	Cu *K*α
μ (mm^−1^)	2.02
Crystal size (mm)	0.40 × 0.09 × 0.04

Data collection	
Diffractometer	XtaLAB Synergy, Dualflex, HyPix-Arc 100
Absorption correction	Multi-scan (*CrysAlis PRO*; Rigaku OD, 2022 ▸)
*T*_min_, *T*_max_	0.503, 1.000
No. of measured, independent and observed [*I* > 2σ(*I*)] reflections	53004, 5873, 5331
*R*_int_	0.051
(sin θ/λ)_max_ (Å^−1^)	0.634

Refinement	
*R*[*F*^2^ > 2σ(*F*^2^)], *wR*(*F*^2^), *S*	0.059, 0.171, 1.06
No. of reflections	5873
No. of parameters	387
No. of restraints	7
H-atom treatment	H-atom parameters constrained
Δρ_max_, Δρ_min_ (e Å^−3^)	0.83, −0.52

Computer programs: *CrysAlis PRO* (Rigaku OD, 2022 ▸), *SHELXT2018/2* (Sheldrick, 2015*a* ▸), *SHELXL2018/3* (Sheldrick, 2015*b* ▸), *OLEX2* (Dolomanov *et al.*, 2009 ▸), and *DIAMOND* (Brandenburg & Putz, 2025 ▸).

## supplementary crystallographic information

### 4-(3,5-Dimethyl-1,7-diphenyl-1,7-dihydrodipyrazolo[3,4-b:4',3'-e]pyridin-4-yl)phenol dimethyl sulfoxide disolvate Crystal data

**Table:**

C_27_H_21_N_5_O·2C_2_H_6_OS	*Z* = 2
*M**_r_* = 587.74	*F*(000) = 620
Triclinic, *P*1	*D*_x_ = 1.354 Mg m^−^^3^
*a* = 7.5736 (1) Å	Cu *K*α radiation, λ = 1.54184 Å
*b* = 12.0242 (1) Å	Cell parameters from 34222 reflections
*c* = 16.1594 (2) Å	θ = 5.6–77.6°
α = 91.824 (1)°	µ = 2.02 mm^−^^1^
β = 100.953 (1)°	*T* = 100 K
γ = 92.657 (1)°	Needle, colourless
*V* = 1441.98 (3) Å^3^	0.40 × 0.09 × 0.04 mm

### 4-(3,5-Dimethyl-1,7-diphenyl-1,7-dihydrodipyrazolo[3,4-b:4',3'-e]pyridin-4-yl)phenol dimethyl sulfoxide disolvate Data collection

**Table:**

XtaLAB Synergy, Dualflex, HyPix-Arc 100 diffractometer	5873 independent reflections
Radiation source: micro-focus sealed X-ray tube, PhotonJet (Cu) X-ray Source	5331 reflections with *I* > 2σ(*I*)
Mirror monochromator	*R*_int_ = 0.051
Detector resolution: 10.0000 pixels mm^-1^	θ_max_ = 77.9°, θ_min_ = 5.6°
ω scans	*h* = −9→8
Absorption correction: multi-scan (CrysAlisPro; Rigaku OD, 2022)	*k* = −14→15
*T*_min_ = 0.503, *T*_max_ = 1.000	*l* = −19→20
53004 measured reflections	

### 4-(3,5-Dimethyl-1,7-diphenyl-1,7-dihydrodipyrazolo[3,4-b:4',3'-e]pyridin-4-yl)phenol dimethyl sulfoxide disolvate Refinement

**Table:**

Refinement on *F*^2^	Primary atom site location: dual
Least-squares matrix: full	Hydrogen site location: inferred from neighbouring sites
*R*[*F*^2^ > 2σ(*F*^2^)] = 0.059	H-atom parameters constrained
*wR*(*F*^2^) = 0.171	*w* = 1/[σ^2^(*F*_o_^2^) + (0.1271*P*)^2^ + 0.4936*P*] where *P* = (*F*_o_^2^ + 2*F*_c_^2^)/3
*S* = 1.06	(Δ/σ)_max_ < 0.001
5873 reflections	Δρ_max_ = 0.83 e Å^−^^3^
387 parameters	Δρ_min_ = −0.52 e Å^−^^3^
7 restraints	

### 4-(3,5-Dimethyl-1,7-diphenyl-1,7-dihydrodipyrazolo[3,4-b:4',3'-e]pyridin-4-yl)phenol dimethyl sulfoxide disolvate Special details

**Table:**

Geometry. All esds (except the esd in the dihedral angle between two l.s. planes) are estimated using the full covariance matrix. The cell esds are taken into account individually in the estimation of esds in distances, angles and torsion angles; correlations between esds in cell parameters are only used when they are defined by crystal symmetry. An approximate (isotropic) treatment of cell esds is used for estimating esds involving l.s. planes.

### 4-(3,5-Dimethyl-1,7-diphenyl-1,7-dihydrodipyrazolo[3,4-b:4',3'-e]pyridin-4-yl)phenol dimethyl sulfoxide disolvate Fractional atomic coordinates and isotropic or equivalent isotropic displacement parameters (Å^2^)

**Table:**

	*x*	*y*	*z*	*U*_iso_*/*U*_eq_	Occ. (<1)
S1	0.30701 (7)	0.22453 (4)	0.99930 (3)	0.03382 (17)	
O3	0.3487 (2)	0.28941 (14)	0.92642 (9)	0.0404 (4)	
C28	0.2308 (3)	0.3217 (2)	1.06824 (16)	0.0489 (6)	
H28A	0.329852	0.375409	1.092503	0.073*	
H28B	0.189338	0.282048	1.113635	0.073*	
H28C	0.131123	0.361337	1.036545	0.073*	
C29	0.0947 (3)	0.1519 (2)	0.96113 (14)	0.0436 (5)	
H29A	0.005564	0.205230	0.938663	0.065*	
H29B	0.055373	0.113158	1.007445	0.065*	
H29C	0.106705	0.097539	0.916327	0.065*	
S2B	0.3600 (11)	0.0684 (7)	0.1856 (4)	0.068 (3)	0.077 (2)
O2	0.1632 (2)	0.06089 (13)	0.15533 (10)	0.0392 (4)	
C30	0.4071 (3)	0.0163 (2)	0.28850 (14)	0.0401 (5)	
H30D	0.302046	−0.028140	0.298583	0.060*	0.077 (2)
H30E	0.434494	0.078689	0.330174	0.060*	0.077 (2)
H30F	0.510854	−0.030569	0.293577	0.060*	0.077 (2)
H30A	0.474427	0.085736	0.281302	0.060*	0.923 (2)
H30B	0.491644	−0.039993	0.310225	0.060*	0.923 (2)
H30C	0.327311	0.029805	0.328486	0.060*	0.923 (2)
C31	0.4576 (3)	−0.0358 (2)	0.13218 (16)	0.0459 (6)	
H31D	0.370032	−0.098893	0.115536	0.069*	0.077 (2)
H31E	0.564391	−0.061065	0.169611	0.069*	0.077 (2)
H31F	0.492617	−0.005288	0.081776	0.069*	0.077 (2)
H31A	0.409674	−0.059790	0.073354	0.069*	0.923 (2)
H31B	0.545261	−0.088296	0.157723	0.069*	0.923 (2)
H31C	0.516730	0.038740	0.134179	0.069*	0.923 (2)
O1	0.6914 (2)	0.32737 (14)	0.91211 (9)	0.0390 (4)	
H1	0.584381	0.310132	0.915665	0.059*	
N1	0.69150 (19)	0.23346 (12)	0.41144 (9)	0.0221 (3)	
N2	0.7253 (2)	0.33241 (12)	0.37466 (9)	0.0212 (3)	
N3	0.78724 (19)	0.52537 (12)	0.41716 (9)	0.0200 (3)	
N4	0.82744 (19)	0.70547 (12)	0.49050 (9)	0.0209 (3)	
N5	0.8160 (2)	0.74841 (12)	0.56996 (9)	0.0228 (3)	
C1	0.6986 (3)	0.35225 (16)	0.83146 (11)	0.0283 (4)	
C2	0.5578 (2)	0.32194 (15)	0.76418 (11)	0.0255 (4)	
H2	0.453109	0.281988	0.774056	0.031*	
C3	0.5709 (2)	0.35011 (14)	0.68320 (11)	0.0218 (3)	
H3	0.475339	0.328080	0.637669	0.026*	
C4	0.7221 (2)	0.41044 (14)	0.66714 (11)	0.0207 (3)	
C5	0.8633 (2)	0.43868 (15)	0.73544 (11)	0.0237 (4)	
H5	0.967841	0.479059	0.725874	0.028*	
C6	0.8530 (3)	0.40889 (16)	0.81619 (11)	0.0275 (4)	
H6	0.951264	0.427000	0.861367	0.033*	
C7	0.7347 (2)	0.44764 (14)	0.58206 (10)	0.0192 (3)	
C8	0.7176 (2)	0.37525 (14)	0.51013 (10)	0.0194 (3)	
C9	0.6869 (2)	0.25707 (14)	0.49090 (11)	0.0212 (3)	
C10	0.6516 (2)	0.16418 (14)	0.54567 (11)	0.0243 (4)	
H10A	0.530716	0.168890	0.558458	0.036*	
H10B	0.741089	0.169984	0.598313	0.036*	
H10C	0.659929	0.092663	0.516209	0.036*	
C11	0.7439 (2)	0.42006 (14)	0.43258 (10)	0.0197 (3)	
C12	0.7968 (2)	0.59164 (14)	0.48631 (10)	0.0193 (3)	
C13	0.7707 (2)	0.56000 (14)	0.56730 (10)	0.0198 (3)	
C14	0.7831 (2)	0.66405 (14)	0.61538 (11)	0.0219 (4)	
C15	0.7561 (3)	0.68638 (15)	0.70355 (11)	0.0263 (4)	
H15A	0.853542	0.655118	0.743345	0.039*	
H15B	0.640259	0.651681	0.710256	0.039*	
H15C	0.756755	0.766976	0.714839	0.039*	
C16	0.7378 (2)	0.33181 (14)	0.28846 (11)	0.0215 (4)	
C17	0.7958 (3)	0.23643 (15)	0.25179 (12)	0.0266 (4)	
H17	0.826235	0.173435	0.284544	0.032*	
C18	0.8083 (3)	0.23475 (17)	0.16735 (12)	0.0309 (4)	
H18	0.846711	0.169958	0.142210	0.037*	
C19	0.7653 (3)	0.32672 (18)	0.11898 (12)	0.0307 (4)	
H19	0.775542	0.325209	0.061267	0.037*	
C20	0.7073 (3)	0.42092 (17)	0.15576 (12)	0.0291 (4)	
H20	0.677766	0.483941	0.122899	0.035*	
C21	0.6921 (2)	0.42377 (15)	0.24026 (11)	0.0250 (4)	
H21	0.650837	0.488008	0.264866	0.030*	
C22	0.8558 (2)	0.78004 (14)	0.42752 (11)	0.0206 (3)	
C23	0.8872 (2)	0.74094 (14)	0.35001 (11)	0.0239 (4)	
H23	0.890289	0.663167	0.338480	0.029*	
C24	0.9139 (3)	0.81630 (15)	0.28935 (12)	0.0266 (4)	
H24	0.935191	0.789373	0.236398	0.032*	
C25	0.9100 (2)	0.93004 (15)	0.30511 (12)	0.0263 (4)	
H25	0.928651	0.981009	0.263482	0.032*	
C26	0.8782 (3)	0.96852 (15)	0.38298 (12)	0.0282 (4)	
H26	0.875491	1.046330	0.394494	0.034*	
C27	0.8504 (2)	0.89423 (15)	0.44383 (12)	0.0249 (4)	
H27	0.827706	0.921163	0.496514	0.030*	
S2A	0.27650 (7)	−0.03230 (4)	0.18957 (3)	0.0311 (2)	0.923 (2)

### 4-(3,5-Dimethyl-1,7-diphenyl-1,7-dihydrodipyrazolo[3,4-b:4',3'-e]pyridin-4-yl)phenol dimethyl sulfoxide disolvate Atomic displacement parameters (Å^2^)

**Table:**

	*U*^11^	*U*^22^	*U*^33^	*U*^12^	*U*^13^	*U*^23^
S1	0.0292 (3)	0.0456 (3)	0.0294 (3)	−0.0002 (2)	0.0125 (2)	0.0054 (2)
O3	0.0404 (9)	0.0513 (9)	0.0348 (8)	−0.0005 (7)	0.0204 (7)	0.0073 (7)
C28	0.0405 (13)	0.0682 (16)	0.0404 (12)	−0.0102 (11)	0.0199 (10)	−0.0153 (11)
C29	0.0425 (13)	0.0502 (13)	0.0367 (11)	−0.0112 (10)	0.0071 (9)	0.0057 (9)
S2B	0.090 (8)	0.054 (5)	0.070 (6)	0.017 (5)	0.036 (5)	0.020 (4)
O2	0.0324 (8)	0.0449 (8)	0.0410 (8)	0.0102 (6)	0.0057 (6)	0.0068 (6)
C30	0.0325 (11)	0.0456 (12)	0.0416 (11)	0.0012 (9)	0.0047 (9)	0.0100 (9)
C31	0.0444 (13)	0.0469 (13)	0.0530 (14)	0.0112 (10)	0.0232 (11)	0.0073 (10)
O1	0.0341 (8)	0.0606 (10)	0.0244 (7)	−0.0034 (7)	0.0108 (6)	0.0096 (6)
N1	0.0204 (7)	0.0212 (7)	0.0262 (7)	0.0000 (5)	0.0083 (6)	0.0038 (5)
N2	0.0216 (7)	0.0212 (7)	0.0227 (7)	0.0003 (5)	0.0086 (5)	0.0029 (5)
N3	0.0176 (7)	0.0211 (7)	0.0230 (7)	0.0011 (5)	0.0076 (5)	0.0029 (5)
N4	0.0197 (7)	0.0215 (7)	0.0233 (7)	0.0002 (5)	0.0084 (5)	0.0020 (5)
N5	0.0214 (7)	0.0245 (7)	0.0238 (7)	0.0008 (6)	0.0082 (6)	−0.0001 (5)
C1	0.0282 (10)	0.0352 (9)	0.0244 (9)	0.0044 (8)	0.0106 (7)	0.0065 (7)
C2	0.0205 (9)	0.0300 (9)	0.0286 (9)	0.0014 (7)	0.0105 (7)	0.0056 (7)
C3	0.0167 (8)	0.0250 (8)	0.0253 (8)	0.0029 (6)	0.0073 (6)	0.0030 (6)
C4	0.0182 (8)	0.0227 (8)	0.0230 (8)	0.0029 (6)	0.0077 (6)	0.0029 (6)
C5	0.0192 (8)	0.0266 (8)	0.0268 (8)	0.0000 (6)	0.0080 (7)	0.0018 (6)
C6	0.0231 (9)	0.0345 (9)	0.0248 (9)	0.0018 (7)	0.0043 (7)	0.0013 (7)
C7	0.0114 (7)	0.0240 (8)	0.0236 (8)	0.0023 (6)	0.0061 (6)	0.0037 (6)
C8	0.0133 (7)	0.0228 (8)	0.0239 (8)	0.0009 (6)	0.0078 (6)	0.0040 (6)
C9	0.0169 (8)	0.0228 (8)	0.0253 (8)	0.0015 (6)	0.0068 (6)	0.0034 (6)
C10	0.0239 (9)	0.0236 (8)	0.0274 (8)	0.0003 (7)	0.0099 (7)	0.0043 (7)
C11	0.0153 (7)	0.0225 (8)	0.0221 (8)	0.0016 (6)	0.0058 (6)	0.0024 (6)
C12	0.0128 (7)	0.0223 (8)	0.0241 (8)	0.0006 (6)	0.0063 (6)	0.0031 (6)
C13	0.0142 (7)	0.0237 (8)	0.0228 (8)	0.0014 (6)	0.0068 (6)	0.0021 (6)
C14	0.0178 (8)	0.0247 (8)	0.0246 (8)	0.0014 (6)	0.0070 (6)	0.0018 (6)
C15	0.0267 (9)	0.0280 (8)	0.0267 (9)	0.0008 (7)	0.0119 (7)	−0.0010 (7)
C16	0.0166 (8)	0.0253 (8)	0.0234 (8)	−0.0015 (6)	0.0066 (6)	−0.0004 (6)
C17	0.0262 (9)	0.0276 (9)	0.0280 (9)	0.0017 (7)	0.0101 (7)	0.0005 (7)
C18	0.0292 (10)	0.0357 (10)	0.0295 (9)	−0.0002 (8)	0.0114 (8)	−0.0041 (8)
C19	0.0257 (9)	0.0450 (11)	0.0226 (8)	−0.0037 (8)	0.0093 (7)	0.0001 (7)
C20	0.0256 (9)	0.0358 (10)	0.0263 (9)	−0.0026 (7)	0.0059 (7)	0.0063 (7)
C21	0.0213 (9)	0.0291 (9)	0.0253 (8)	−0.0002 (7)	0.0063 (7)	0.0024 (7)
C22	0.0133 (7)	0.0236 (8)	0.0262 (8)	0.0003 (6)	0.0068 (6)	0.0049 (6)
C23	0.0213 (8)	0.0230 (8)	0.0293 (9)	−0.0004 (6)	0.0099 (7)	0.0020 (7)
C24	0.0241 (9)	0.0297 (9)	0.0279 (9)	0.0003 (7)	0.0100 (7)	0.0037 (7)
C25	0.0225 (9)	0.0271 (8)	0.0305 (9)	0.0007 (7)	0.0074 (7)	0.0085 (7)
C26	0.0300 (10)	0.0223 (8)	0.0334 (10)	0.0026 (7)	0.0084 (8)	0.0039 (7)
C27	0.0235 (9)	0.0242 (8)	0.0281 (9)	0.0027 (6)	0.0071 (7)	0.0019 (7)
S2A	0.0253 (3)	0.0306 (3)	0.0386 (3)	0.0008 (2)	0.0087 (2)	0.0047 (2)

### 4-(3,5-Dimethyl-1,7-diphenyl-1,7-dihydrodipyrazolo[3,4-b:4',3'-e]pyridin-4-yl)phenol dimethyl sulfoxide disolvate Geometric parameters (Å, º)

**Table:**

S1—O3	1.5073 (15)	C3—H3	0.9500
S1—C28	1.779 (2)	C3—C4	1.398 (3)
S1—C29	1.785 (2)	C4—C5	1.404 (2)
C28—H28A	0.9800	C4—C7	1.478 (2)
C28—H28B	0.9800	C5—H5	0.9500
C28—H28C	0.9800	C5—C6	1.379 (3)
C29—H29A	0.9800	C6—H6	0.9500
C29—H29B	0.9800	C7—C8	1.412 (2)
C29—H29C	0.9800	C7—C13	1.403 (2)
S2B—O2	1.476 (8)	C8—C9	1.444 (2)
S2B—C30	1.774 (6)	C8—C11	1.424 (2)
S2B—C31	1.766 (7)	C9—C10	1.492 (2)
O2—S2A	1.4989 (15)	C10—H10A	0.9800
C30—H30D	0.9800	C10—H10B	0.9800
C30—H30E	0.9800	C10—H10C	0.9800
C30—H30F	0.9800	C12—C13	1.421 (2)
C30—H30A	0.9800	C13—C14	1.441 (2)
C30—H30B	0.9800	C14—C15	1.495 (2)
C30—H30C	0.9800	C15—H15A	0.9800
C30—S2A	1.778 (2)	C15—H15B	0.9800
C31—H31D	0.9800	C15—H15C	0.9800
C31—H31E	0.9800	C16—C17	1.400 (2)
C31—H31F	0.9800	C16—C21	1.392 (2)
C31—H31A	0.9800	C17—H17	0.9500
C31—H31B	0.9800	C17—C18	1.385 (3)
C31—H31C	0.9800	C18—H18	0.9500
C31—S2A	1.797 (2)	C18—C19	1.390 (3)
O1—H1	0.8400	C19—H19	0.9500
O1—C1	1.357 (2)	C19—C20	1.390 (3)
N1—N2	1.3815 (19)	C20—H20	0.9500
N1—C9	1.314 (2)	C20—C21	1.391 (3)
N2—C11	1.371 (2)	C21—H21	0.9500
N2—C16	1.414 (2)	C22—C23	1.390 (2)
N3—C11	1.336 (2)	C22—C27	1.394 (2)
N3—C12	1.340 (2)	C23—H23	0.9500
N4—N5	1.3880 (19)	C23—C24	1.392 (2)
N4—C12	1.375 (2)	C24—H24	0.9500
N4—C22	1.418 (2)	C24—C25	1.386 (3)
N5—C14	1.313 (2)	C25—H25	0.9500
C1—C2	1.396 (3)	C25—C26	1.394 (3)
C1—C6	1.392 (3)	C26—H26	0.9500
C2—H2	0.9500	C26—C27	1.388 (3)
C2—C3	1.382 (2)	C27—H27	0.9500
			
O3—S1—C28	106.42 (12)	C13—C7—C4	121.55 (15)
O3—S1—C29	105.77 (10)	C13—C7—C8	114.37 (15)
C28—S1—C29	97.04 (12)	C7—C8—C9	136.98 (16)
S1—C28—H28A	109.5	C7—C8—C11	118.89 (15)
S1—C28—H28B	109.5	C11—C8—C9	104.04 (14)
S1—C28—H28C	109.5	N1—C9—C8	110.94 (14)
H28A—C28—H28B	109.5	N1—C9—C10	118.63 (15)
H28A—C28—H28C	109.5	C8—C9—C10	130.43 (15)
H28B—C28—H28C	109.5	C9—C10—H10A	109.5
S1—C29—H29A	109.5	C9—C10—H10B	109.5
S1—C29—H29B	109.5	C9—C10—H10C	109.5
S1—C29—H29C	109.5	H10A—C10—H10B	109.5
H29A—C29—H29B	109.5	H10A—C10—H10C	109.5
H29A—C29—H29C	109.5	H10B—C10—H10C	109.5
H29B—C29—H29C	109.5	N2—C11—C8	106.84 (14)
O2—S2B—C30	108.5 (4)	N3—C11—N2	124.68 (15)
O2—S2B—C31	109.0 (5)	N3—C11—C8	128.41 (15)
C31—S2B—C30	98.6 (4)	N3—C12—N4	125.47 (15)
S2B—C30—H30D	109.5	N3—C12—C13	127.44 (15)
S2B—C30—H30E	109.5	N4—C12—C13	107.05 (14)
S2B—C30—H30F	109.5	C7—C13—C12	120.07 (15)
H30D—C30—H30E	109.5	C7—C13—C14	135.79 (16)
H30D—C30—H30F	109.5	C12—C13—C14	104.08 (15)
H30E—C30—H30F	109.5	N5—C14—C13	111.18 (15)
H30A—C30—H30B	109.5	N5—C14—C15	119.08 (15)
H30A—C30—H30C	109.5	C13—C14—C15	129.67 (16)
H30B—C30—H30C	109.5	C14—C15—H15A	109.5
S2A—C30—H30A	109.5	C14—C15—H15B	109.5
S2A—C30—H30B	109.5	C14—C15—H15C	109.5
S2A—C30—H30C	109.5	H15A—C15—H15B	109.5
S2B—C31—H31D	109.5	H15A—C15—H15C	109.5
S2B—C31—H31E	109.5	H15B—C15—H15C	109.5
S2B—C31—H31F	109.5	C17—C16—N2	118.88 (16)
H31D—C31—H31E	109.5	C21—C16—N2	120.89 (15)
H31D—C31—H31F	109.5	C21—C16—C17	120.22 (16)
H31E—C31—H31F	109.5	C16—C17—H17	120.3
H31A—C31—H31B	109.5	C18—C17—C16	119.42 (17)
H31A—C31—H31C	109.5	C18—C17—H17	120.3
H31B—C31—H31C	109.5	C17—C18—H18	119.6
S2A—C31—H31A	109.5	C17—C18—C19	120.79 (18)
S2A—C31—H31B	109.5	C19—C18—H18	119.6
S2A—C31—H31C	109.5	C18—C19—H19	120.3
C1—O1—H1	109.5	C18—C19—C20	119.44 (18)
C9—N1—N2	107.49 (14)	C20—C19—H19	120.3
N1—N2—C16	119.75 (14)	C19—C20—H20	119.7
C11—N2—N1	110.67 (14)	C19—C20—C21	120.61 (18)
C11—N2—C16	129.57 (15)	C21—C20—H20	119.7
C11—N3—C12	110.69 (14)	C16—C21—H21	120.2
N5—N4—C22	118.97 (14)	C20—C21—C16	119.50 (17)
C12—N4—N5	110.26 (13)	C20—C21—H21	120.2
C12—N4—C22	130.64 (15)	C23—C22—N4	121.06 (15)
C14—N5—N4	107.39 (14)	C23—C22—C27	119.85 (16)
O1—C1—C2	122.12 (18)	C27—C22—N4	119.08 (16)
O1—C1—C6	118.24 (17)	C22—C23—H23	120.2
C6—C1—C2	119.63 (17)	C22—C23—C24	119.68 (16)
C1—C2—H2	120.0	C24—C23—H23	120.2
C3—C2—C1	119.95 (17)	C23—C24—H24	119.5
C3—C2—H2	120.0	C25—C24—C23	120.94 (17)
C2—C3—H3	119.4	C25—C24—H24	119.5
C2—C3—C4	121.17 (16)	C24—C25—H25	120.5
C4—C3—H3	119.4	C24—C25—C26	119.00 (17)
C3—C4—C5	117.97 (16)	C26—C25—H25	120.5
C3—C4—C7	122.29 (15)	C25—C26—H26	119.7
C5—C4—C7	119.72 (15)	C27—C26—C25	120.62 (17)
C4—C5—H5	119.4	C27—C26—H26	119.7
C6—C5—C4	121.24 (17)	C22—C27—H27	120.1
C6—C5—H5	119.4	C26—C27—C22	119.90 (17)
C1—C6—H6	120.0	C26—C27—H27	120.1
C5—C6—C1	119.97 (16)	O2—S2A—C30	107.23 (10)
C5—C6—H6	120.0	O2—S2A—C31	106.38 (10)
C8—C7—C4	124.05 (15)	C30—S2A—C31	97.34 (12)
			
O1—C1—C2—C3	179.40 (17)	C7—C8—C11—N3	1.2 (3)
O1—C1—C6—C5	−178.02 (17)	C7—C13—C14—N5	−178.12 (18)
N1—N2—C11—N3	176.07 (15)	C7—C13—C14—C15	−1.2 (3)
N1—N2—C11—C8	−1.05 (18)	C8—C7—C13—C12	−3.5 (2)
N1—N2—C16—C17	−28.3 (2)	C8—C7—C13—C14	173.18 (18)
N1—N2—C16—C21	151.19 (16)	C9—N1—N2—C11	0.57 (18)
N2—N1—C9—C8	0.16 (18)	C9—N1—N2—C16	179.90 (14)
N2—N1—C9—C10	179.80 (14)	C9—C8—C11—N2	1.07 (17)
N2—C16—C17—C18	179.88 (16)	C9—C8—C11—N3	−175.91 (16)
N2—C16—C21—C20	179.48 (16)	C11—N2—C16—C17	150.91 (18)
N3—C12—C13—C7	1.7 (3)	C11—N2—C16—C21	−29.6 (3)
N3—C12—C13—C14	−175.87 (16)	C11—N3—C12—N4	−175.80 (15)
N4—N5—C14—C13	−0.10 (19)	C11—N3—C12—C13	1.5 (2)
N4—N5—C14—C15	−177.42 (14)	C11—C8—C9—N1	−0.77 (18)
N4—C12—C13—C7	179.44 (14)	C11—C8—C9—C10	179.64 (16)
N4—C12—C13—C14	1.83 (17)	C12—N3—C11—N2	−179.45 (15)
N4—C22—C23—C24	179.77 (15)	C12—N3—C11—C8	−3.0 (2)
N4—C22—C27—C26	179.89 (15)	C12—N4—N5—C14	1.33 (18)
N5—N4—C12—N3	175.74 (15)	C12—N4—C22—C23	−11.4 (3)
N5—N4—C12—C13	−2.01 (17)	C12—N4—C22—C27	168.02 (16)
N5—N4—C22—C23	173.13 (15)	C12—C13—C14—N5	−1.08 (19)
N5—N4—C22—C27	−7.5 (2)	C12—C13—C14—C15	175.87 (17)
C1—C2—C3—C4	−1.1 (3)	C13—C7—C8—C9	178.09 (18)
C2—C1—C6—C5	2.6 (3)	C13—C7—C8—C11	2.2 (2)
C2—C3—C4—C5	2.0 (3)	C16—N2—C11—N3	−3.2 (3)
C2—C3—C4—C7	−176.14 (16)	C16—N2—C11—C8	179.71 (15)
C3—C4—C5—C6	−0.6 (3)	C16—C17—C18—C19	0.5 (3)
C3—C4—C7—C8	−57.1 (2)	C17—C16—C21—C20	−1.1 (3)
C3—C4—C7—C13	124.85 (18)	C17—C18—C19—C20	−0.7 (3)
C4—C5—C6—C1	−1.7 (3)	C18—C19—C20—C21	0.0 (3)
C4—C7—C8—C9	−0.1 (3)	C19—C20—C21—C16	0.8 (3)
C4—C7—C8—C11	−175.98 (14)	C21—C16—C17—C18	0.4 (3)
C4—C7—C13—C12	174.76 (14)	C22—N4—N5—C14	177.67 (14)
C4—C7—C13—C14	−8.6 (3)	C22—N4—C12—N3	0.0 (3)
C5—C4—C7—C8	124.86 (18)	C22—N4—C12—C13	−177.79 (15)
C5—C4—C7—C13	−53.2 (2)	C22—C23—C24—C25	0.1 (3)
C6—C1—C2—C3	−1.3 (3)	C23—C22—C27—C26	−0.7 (3)
C7—C4—C5—C6	177.57 (16)	C23—C24—C25—C26	−0.2 (3)
C7—C8—C9—N1	−177.03 (18)	C24—C25—C26—C27	−0.2 (3)
C7—C8—C9—C10	3.4 (3)	C25—C26—C27—C22	0.6 (3)
C7—C8—C11—N2	178.16 (14)	C27—C22—C23—C24	0.4 (3)

### 4-(3,5-Dimethyl-1,7-diphenyl-1,7-dihydrodipyrazolo[3,4-b:4',3'-e]pyridin-4-yl)phenol dimethyl sulfoxide disolvate Hydrogen-bond geometry (Å, º)

**Table:**

*D*—H···*A*	*D*—H	H···*A*	*D*···*A*	*D*—H···*A*
C19—H19···O1^i^	0.95	2.37	3.283 (2)	160
C29—H29*B*···O2^ii^	0.98	2.48	3.312 (3)	142

Symmetry codes: (i) *x*, *y*, *z*−1; (ii) *x*, *y*, *z*+1.
